# Osteoprotegerin (OPG): A potential biomarker for adverse cardiovascular events in stable coronary artery disease

**DOI:** 10.1002/hsr2.2253

**Published:** 2024-07-22

**Authors:** Syed Muhammad Muneeb Akhtar, Azzam Ali, Areeba Fareed, Jina Hasan

**Affiliations:** ^1^ Department of Medicine Dow University of Health Sciences Karachi Pakistan; ^2^ Karachi Medical and Dental College Karachi Pakistan; ^3^ Faculty of Medical Sciences Lebanese University Beirut Lebanon

**Keywords:** atherosclerosis, biomarkers, cardiovascular events, coronary artery disease (CAD), osteoprotegerin (OPG)

## Abstract

**Background:**

Osteoprotegerin (OPG) is a secretory glycoprotein known for its involvement in bone metabolism and immune regulation. Research has extended OPG's significance in cardiovascular diseases (CVDs). Elevated OPG levels have been associated with increased cardiovascular risks, prompting interest in its role as a potential biomarker.

**Main Body:**

This study summarizes several studies that investigated the relationship between OPG levels and the incidence of CVD. The studies indicate that higher plasma levels of OPG are associated with an increased incidence of all‐cause death, cardiovascular death, and heart failure, even after adjusting for clinical confounders. Moreover, the findings suggest that OPG has the potential to serve as a predictive biomarker for adverse cardiovascular events in the patient population studied. The findings suggest that OPG could aid in risk stratification, allowing clinicians to identify high‐risk patients who might benefit from intensified preventive measures or tailored therapeutic interventions. Therefore, early identification of individuals at risk for adverse cardiovascular events could lead to improved patient outcomes and reduced disease burden.

**Conclusions:**

OPG's role in bone health and immune regulation has expanded to potential use as a biomarker for adverse cardiovascular events in stable coronary artery disease (CAD) patients. Despite limitations, its association with cardiovascular risks highlights its importance in risk assessment and personalized interventions.

## 1 BACKGROUND

Osteoprotegerin (OPG), a secretory glycoprotein, is known to be a key player in bone metabolism and immune regulation.^1^, ^2^ OPG mainly acts as a regulator of bone remodeling in normal and clinical settings. It impedes the interaction between the receptor activator of nuclear factor kappa B (RANK) and its ligand RANKL by acting as a “decoy receptor.”^3^, ^4^ RANKL is a significant activator of osteoclast differentiation and maturation, which in turn promotes osteoclastogenesis. By blocking the connection between RANKL and the RANK receptor, OPG inhibits osteoclastogenesis and osteoclast maturation, thereby preventing osteolysis processes.^4^, ^5^ However, research has also uncovered its significance beyond bone health, shedding light on its involvement in cardiovascular diseases (CVDs). Atherosclerosis is characterized by plaque accumulation within arteries and is the principal cause of CVDs; including coronary artery disease (CAD), which is the third‐leading cause of mortality worldwide and is associated with 17.8 million deaths annually.^6^ OPG and RANKL have both been identified in atherosclerotic lesions.^7^ The increased concentration of OPG in CAD, during acute coronary syndromes, is considered a strong indicator of the risk of adverse cardiovascular events and a poor prognosis.^8^, ^9^ Clinical studies have also shown that patients with unstable angina (UA) and acute myocardial infarction have higher circulating levels of OPG, while patients with stable angina or established coronary disease do not, indicating that the OPG/RANK/RANKL system may play a significant role in plaque stability.^10^ Several studies have confirmed that high plasma levels of OPG and low levels of TNF‐related apoptosis‐inducing ligand (TRAIL), along with a high OPG/TRAIL ratio, are strong predictors of a poor prognosis for patients with myocardial infarction. Furthermore, high levels of OPG and a high OPG/TRAIL ratio during the acute phase of myocardial infarction are predominantly indicative of adverse left ventricular remodeling and postinfarction heart failure (HF).^11^ Additionally, high concentrations of OPG are highly associated with a higher frequency of hospitalizations due to exacerbated ischemic HF with a reduced ejection fraction.^4^ Significant changes in OPG plasma concentrations have been observed in patients with UA and in patients with acute myocardial infarction.^8^, ^9^ Thus, the intricate interplay between OPG and cardiovascular health has sparked interest in investigating OPG as a potential biomarker for cardiovascular risk assessment.

## 2 MAIN BODY

A study conducted by Ma et al.^12^ has investigated the relationship between plasma OPG level and the prognosis of patients with stable angina. The study included 3766 patients with CAD, and the median OPG concentration was found to be 2.2 ng/mL. It was found that higher plasma levels of OPG were associated with a greater incidence of all‐cause death, cardiovascular death, and HF; even after adjusting for clinical confounders.^12^ In addition to that, Kaplan−Meier analysis demonstrated that patients with the lowest tertiles of OPG levels had a significantly lower possibility of developing clinical events. Moreover, when OPG levels were combined with biomarkers such as NT‐proBNP and cTnT, it led to an improved predictive capability of these markers in patients with stable CAD. Hence, these findings indicate that OPG has the potential to serve as a predictive biomarker for adverse cardiovascular events in this patient population.^12^

In a groundbreaking meta‐analysis led by Tschiderer et al. data amalgamated from nine prospective cohort studies, encompassing over 26,000 participants, unveiled a compelling connection between elevated OPG levels and an 83% augmented risk of CVD. OPG, acting as a surrogate for cumulative exposure to various cardiovascular risk factors, assumes a nuanced mechanistic role with both advantageous and deleterious effects. This study not only sheds light on the intricate interplay between OPG, cardiovascular health, and mortality but also underscores OPG's potential as a reliable biomarker, demonstrating an association strength on par with established cardiovascular indicators.^13^

Simultaneously, Marques et al.'s meticulous study sheds light on the profound implications of elevated OPG levels in patients with chronic kidney disease (CKD). Their findings underscore a significant correlation between heightened OPG concentrations and an increased susceptibility to both all‐cause and cardiovascular mortality. The investigation delves into the intricate associations between OPG and markers of myocardial damage, suggesting potential links to atherosclerotic disease and myocardial ischemia. Despite OPG's recognized involvement in vascular calcification, the precise mechanisms driving OPG‐related mortality in CKD patients remain elusive. Notably, the study unveils OPG as an independent marker for cardiovascular mortality, resilient even to adjustments for inflammatory markers in multivariate analysis. This research contributes robust evidence, emphasizing the elevated OPG levels as a crucial indicator of heightened mortality risk in CKD patients, particularly concerning cardiovascular events.^14^

In a parallel narrative, Qing‐xiu Huang et al.'s comprehensive meta‐analysis corroborates and extends these pivotal insights. The synthesis of multiple studies reveals a substantial correlation between elevated OPG concentrations and an escalated risk of cardiovascular death in CKD patients. The meta‐analysis employs a nuanced approach, considering OPG concentration both categorically and as a continuous variable. This dual perspective not only provides qualitative judgments but also furnishes precise quantitative insights, demonstrating that each incremental 1 pmol/L increase in OPG concentration corresponds to a 4% elevation in the risk of cardiovascular mortality. While the meta‐analysis predominantly focuses on the dialysis population, it conscientiously highlights the imperative for further investigations within the non‐dialysis CKD cohort, thereby broadening the applicability of these crucial findings. The identified association between OPG and vascular calcification serves as a plausible mechanistic link, offering a compelling explanation for the heightened cardiovascular mortality risk observed in CKD patients with elevated OPG levels. These studies significantly advance our understanding of the intricate interplay between OPG, cardiovascular health, and mortality in the context of CKD.^15^

For this reason, OPG, as a biomarker, could aid in risk stratification, allowing clinicians to identify high‐risk patients who might benefit from intensified preventive measures or tailored therapeutic interventions. Consequently, early identification of individuals at risk for adverse cardiovascular events could lead to improved patient outcomes and reduced disease burden.

## 3 LIMITATIONS

While these pieces of research provide persuasive evidence for OPG's potential as a biomarker for adverse cardiovascular events in patients with stable CAD, it is essential to acknowledge some limitations. OPG, a promising biomarker for atherosclerosis, can significantly improve cardiovascular risk prediction; however, the lack of standardized assays to measure OPG levels and variability in reference ranges can hinder its consistent clinical use. Therefore, there is an urgent need for standardization of reference ranges to facilitate the widespread adoption of OPG as a biomarker.^16^

Secondly, the concentration of OPG may be mediated by many factors, such as statin usage, which was ignored in the analysis. Statins are widely prescribed to patients with CAD for lipid‐lowering effects, and their usage may influence OPG levels.^17^ Thus, adjusting for statin usage and other potential confounding factors could provide a more accurate assessment of OPG's independent predictive value. Furthermore, sex‐specific differences in OPG levels have been reported in previous research, and accounting for this imbalance in future studies may enhance the generalizability of the findings to both male and female populations.^18^ Further, conducting an in‐depth analysis to discern the relationship between OPG levels and ventricular function holds promise, given the frequent alterations in ventricular dynamics observed in CVDs, presenting a pivotal prognostic indicator. Employ advanced imaging modalities, such as echocardiography or cardiac magnetic resonance imaging, to meticulously scrutinize ventricular parameters in correlation with OPG concentrations.^19^

Simultaneously, an exhaustive investigation into the interplay between OPG and inflammatory processes within CVDs is imperative. It requires additional validation studies to confirm its clinical utility, reliability, reproducibility, and predictive value across diverse patient populations. Large‐scale prospective studies are necessary to establish its effectiveness as a biomarker.

## 4 CONCLUSIONS

In conclusion, OPG, initially identified for its role in bone metabolism, has emerged as a promising biomarker for adverse cardiovascular events in patients with stable CAD. As scientists and clinicians continue to investigate OPG's role in CVDs, we may unlock new avenues for early detection and targeted interventions, ultimately improving patient outcomes in CAD and other cardiovascular conditions. As the need for personalized and precise cardiovascular risk prediction grows, OPG's place in the realm of cardiovascular biomarkers may become increasingly significant. Therefore, by addressing the limitations highlighted in the present research, future studies can offer a more comprehensive understanding of OPG's potential as a biomarker and its role in CVDs management.

## AUTHOR CONTRIBUTIONS

All Authors contributed equally.

## CONFLICT OF INTEREST STATEMENT

The authors declare no conflict of interest.

## ETHICS STATEMENT

All authors accept full responsibility for the work and the conduct of the study, had access to the data, and controlled the decision to publish.

## TRANSPARENCY STATEMENT

The lead author Jina Hasan affirms that this manuscript is an honest, accurate, and transparent account of the study being reported; that no important aspects of the study have been omitted; and that any discrepancies from the study as planned (and, if relevant, registered) have been explained.

## Data Availability

No new data set was generated.
